# A Modified EpiSC Culture Condition Containing a GSK3 Inhibitor Can Support Germline-Competent Pluripotency in Mice

**DOI:** 10.1371/journal.pone.0095329

**Published:** 2014-04-15

**Authors:** Tomoyuki Tsukiyama, Yasuhide Ohinata

**Affiliations:** 1 PRESTO, Japan Science and Technology Agency (JST), Kawaguchi, Saitama, Japan; 2 Laboratory for Pluripotent Stem Cell Studies, RIKEN Center for Developmental Biology (CDB), Kobe, Hyogo, Japan; 3 Life Science Experimental Facility, Department of Biotechnology, Faculty of Life and Environmental Science, University of Yamanashi, Kofu, Yamanashi, Japan; Wellcome Trust Centre for Stem Cell Research, United Kingdom

## Abstract

Embryonic stem cells (ESCs) can contribute to the tissues of chimeric animals, including the germline. By contrast, epiblast stem cells (EpiSCs) barely contribute to chimeras. These two types of cells are established and maintained under different culture conditions. Here, we show that a modified EpiSC culture condition containing the GSK3 inhibitor CHIR99021 can support a germline-competent pluripotent state that is intermediate between ESCs and EpiSCs. When ESCs were cultured under a modified condition containing bFGF, Activin A, and CHIR99021, they converted to intermediate pluripotent stem cells (INTPSCs). These INTPSCs were able to form teratomas *in vivo* and contribute to chimeras by blastocyst injection. We also induced formation of INTPSCs (iINTPSCs) from mouse embryonic fibroblasts by exogenous expression of four reprogramming factors, Oct3/4, Sox2, Klf4, and c-Myc, under the INTPSC culture condition. These iINTPSCs contributed efficiently to chimeras, including the germline, by blastocyst injection. The INTPSCs exhibited several characteristic properties of both ESCs and EpiSCs. Our results suggest that the modified EpiSC culture condition can support growth of cells that meet the most stringent criteria for pluripotency, and that germline-competent pluripotency may depend on the activation state of Wnt signaling.

## Introduction

Pluripotent stem cells can be classified into two categories: naïve pluripotent stem cells, such as mouse embryonic stem cells (ESCs) and induced pluripotent stem cells (iPSCs), and primed pluripotent stem cells, such as mouse epiblast stem cells (EpiSCs) and human ESCs. In mouse, ESCs derived from the inner cell mass (ICM) of blastocyst-stage embryos exhibit compact dome-shaped colony morphology and dependency on the leukemia inhibitory factor (LIF)–Jak/Stat signaling pathway and/or defined chemical compounds (the GSK3 inhibitor CHIR99021 and the Mek/Erk inhibitor PD0325901 [2i]) [1], [2]. Mouse ESCs can differentiate into the three germ layers *in vitro* and *in vivo* and contribute to chimeras, including the germline, by morula aggregation or blastocyst injection. In human as well, naïve pluripotent stem cells have been established in chemically defined medium containing LIF, basic fibroblast growth factor (bFGF), transforming growth factor beta 1 (TGF-β1), and several small-molecule compounds [3], although it remains to be determined whether this culture condition supports the ability of stem cells to contribute to chimeras and the germline in non-rodent mammalian species.

On the other hand, EpiSCs derived from epiblasts of E5.5–6.5 post-implantation embryos and human ESCs derived (like mouse ESCs) from the ICM of blastocyst-stage embryos exhibit flattened monolayer colony morphology and dependency on the bFGF signaling pathway [4], [5]. Although EpiSCs can differentiate into the three germ layers *in vitro* and *in vivo*, they barely contribute to chimeras. However, it is still unknown whether such differences in the ability to contribute to chimeras and the germline are simply due to differences in the signaling pathways on which these two cell types depend. Previous work demonstrated that minor populations of EpiSCs can contribute to chimeras, including the germline [6], suggesting that a modified EpiSC culture condition can support the maintenance of germline-competent pluripotency.

Although Wnt signaling plays important roles in self-renewal and maintenance of pluripotent stem cells, its effects on the pluripotent state remain enigmatic [7]–[9]. Activation of Wnt signaling in ESCs prevents differentiation into EpiSCs. In the presence of Wnt ligands, self-renewal of ESCs is strongly promoted, whereas in the presence of the Wnt signaling inhibitor IWP2, self-renewal of ESCs is suppressed, and the ESCs differentiate to EpiSCs [10]. On the other hand, inhibition of Wnt signaling is critical for maintenance of the undifferentiated state in post-implantation mouse embryos and EpiSCs. EpiSCs in the undifferentiated state have been established in the presence of bFGF, Activin A, and the canonical Wnt-signaling inhibitor XAV939, whereas stimulation of Wnt signaling with CHIR99021 in the presence of bFGF and Activin A has resulted in rapid differentiation of EpiSCs [11]. These studies showed that the responses to Wnt signaling activation or inhibition clearly differ between ESCs and EpiSCs. Furthermore, ESCs are induced to differentiate into EpiSCs by culture in the presence of bFGF and Activin A, a condition that supports self-renewal of EpiSCs [12]. Therefore, we sought to investigate whether culturing ESCs under an EpiSC culture condition in which Wnt signaling is activated would result in maintenance of the naïve state, the primed state, or differentiation.

In this study, we assessed the effect of the GSK3 inhibitor CHIR99021 under the EpiSC culture condition, and found that a modified EpiSC culture condition containing CHIR99021 promoted conversion of ESCs into intermediate pluripotent stem cells (INTPSCs). These INTPSCs formed three-dimensional (3D) colonies, although they were flatter than mouse ESC colonies. Both ESC-derived INTPSCs and somatic cell–derived induced INTPSCs (iINTPSCs) could differentiate into the three germ layers *in vitro* and *in vivo* and contribute to chimeras, including the germline. Thus, our results demonstrate that INTPSCs satisfy the most stringent criteria for pluripotency, and suggest that Wnt signaling plays an important role in naïve pluripotency in the INTPSC culture condition.

## Results

### Conversion of ESCs into INTPSCs under a Modified EpiSC Culture Condition Containing CHIR99021

**Figure 1:**
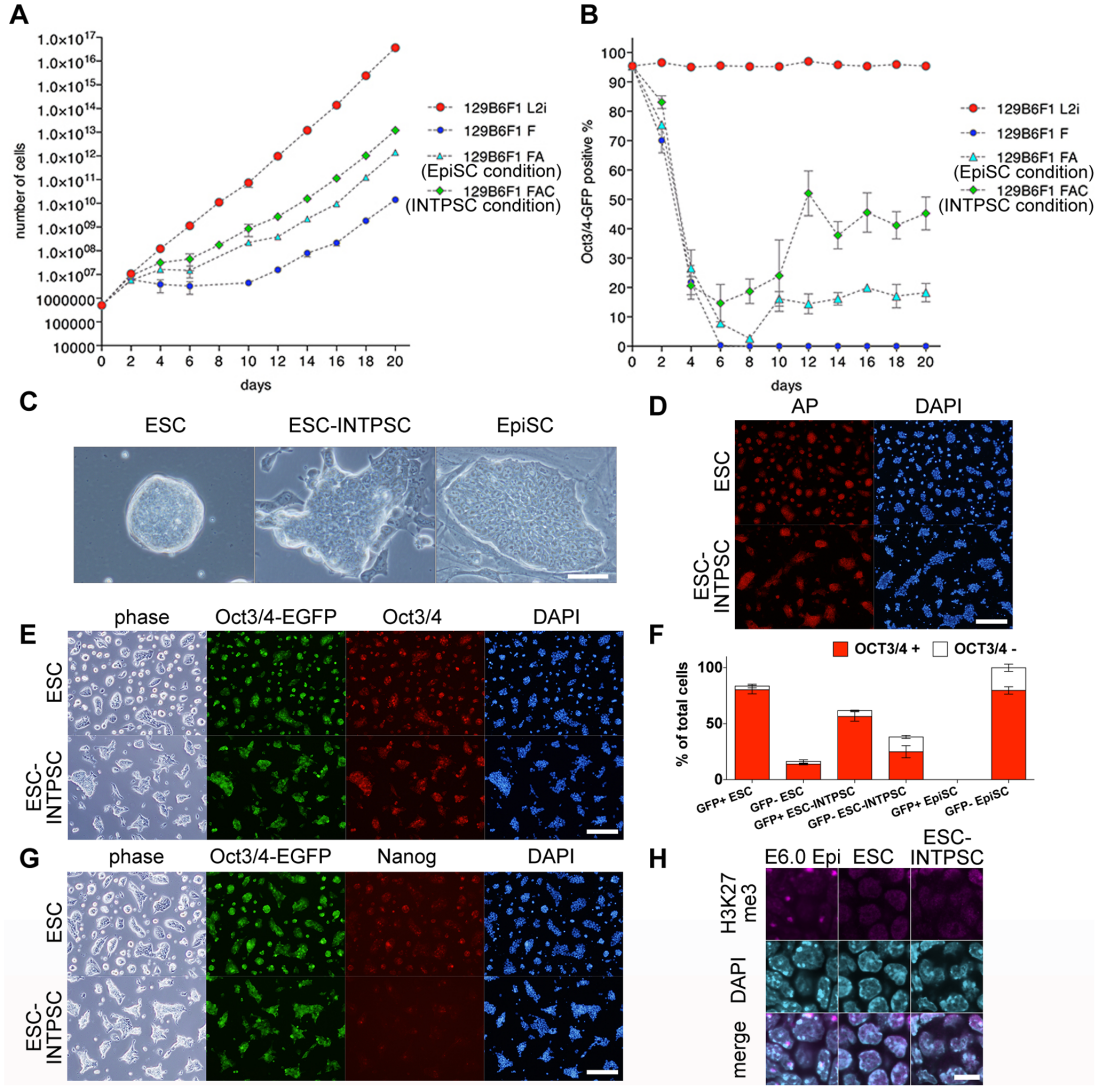
Conversion of ESCs into INTPSCs in a modified EpiSC culture condition containing CHIR99021. (A) Proliferation of ESCs in the presence of LIF/2i, F, FA, and FAC. (B) Proportions of Oct3/4-GFP–positive ESCs in the presence of LIF/2i, F, FA, and FAC. (C) Phase-contrast images of ESCs, ESC-INTPSCs, and EpiSCs. Scale bar, 50 µm. (D) Alkaline-phosphatase staining of ESCs and ESC-INTPSCs. Scale bar, 500 µm. (E and G) Immunostaining of ESCs and ESC-INTPSCs. Scale bar, 500 µm. (F) Percentage of OCT3/4-positive or -negative cells in GOF18 Oct3/4-GFP–positive or –negative ESCs, ESC-INTPSCs, and EpiSCs. (H) Immunostaining for H3K27me3 in E6.0 epiblasts, ESCs, and ESC-INTPSCs. Scale bar, 10 µm.

We first evaluated modified EpiSC culture conditions containing various combinations of 12 ng/ml bFGF (F), 10 ng/ml Activin A (A), and/or 3 µM of the specific GKS3 inhibitor CHIR99021 (C). To this end, we cultured GOF18 ESCs harboring an Oct3/4-GFP reporter in the presence of LIF/2i, F, FA (the EpiSC condition), or FAC (the INTPSC condition) [13]. In the presence of F, these cells proliferated slowly (Fig. 1A); however, the proportion of Oct3/4-GFP–positive cells was greatly reduced after the medium change, and Oct3/4-GFP fluorescence disappeared 6 days later (Fig. 1B). Additionally, in the EpiSC condition, although the cells proliferated more rapidly than in the presence of F (Fig. 1A), the proportion of Oct3/4-GFP–positive cells was greatly reduced after the medium change, and only 10–20% of cells expressed Oct3/4-GFP fluorescence 10 days later (Fig. 1B). By contrast, under the INTPSC condition, the cells proliferated more rapidly than under the EpiSC condition (Fig. 1A), and 40–50% of cells expressed Oct3/4-GFP fluorescence 12 days after the medium change (Fig. 1B). Subsequently, this proportion of Oct3/4-GFP–positive cells was maintained for at least until 30 passages (data not shown).

The resultant cells, which we named ESC-INTPSCs, could propagate by single-cell dissociation and consistently formed 3D colonies, although they were flatter than mouse ESC colonies (Fig. 1C). Immunocytological analysis revealed that these cells were positive for mouse ESC markers, such as OCT3/4 and NANOG (Fig. 1E and G), as well as alkaline phosphatase (Fig. 1D). We found that most ESC-INTPSCs were OCT3/4-positive regardless of whether they were GOF18 Oct3/4-GFP–positive, and in total, 81.22±1.99% of ESC-INTPSCs (>30 passages) were positive for OCT3/4 (Fig. 1F). This ratio was lower than that of ESCs (93.18±5.31%), but comparable to that of EpiSCs (79.80±5.68%), indicating that the INTPSC condition could support the long-term maintenance of undifferentiated cells to a similar extent as the EpiSC condition. Furthermore, in order to investigate epigenetic modifications in these cells, we stained for trimethyl-Histone H3 Lysine27 (H3K27me3); H3K27me3 accumulates on the inactive X chromosome, where it can be detected as a spot. Like ESCs, but unlike E6.0 epiblast cells, ESC-INTPSCs did not contain H3K27me3 spots (Fig. 1H), indicating that the INTPSCs contained two active X chromosomes.

**Figure 2:**
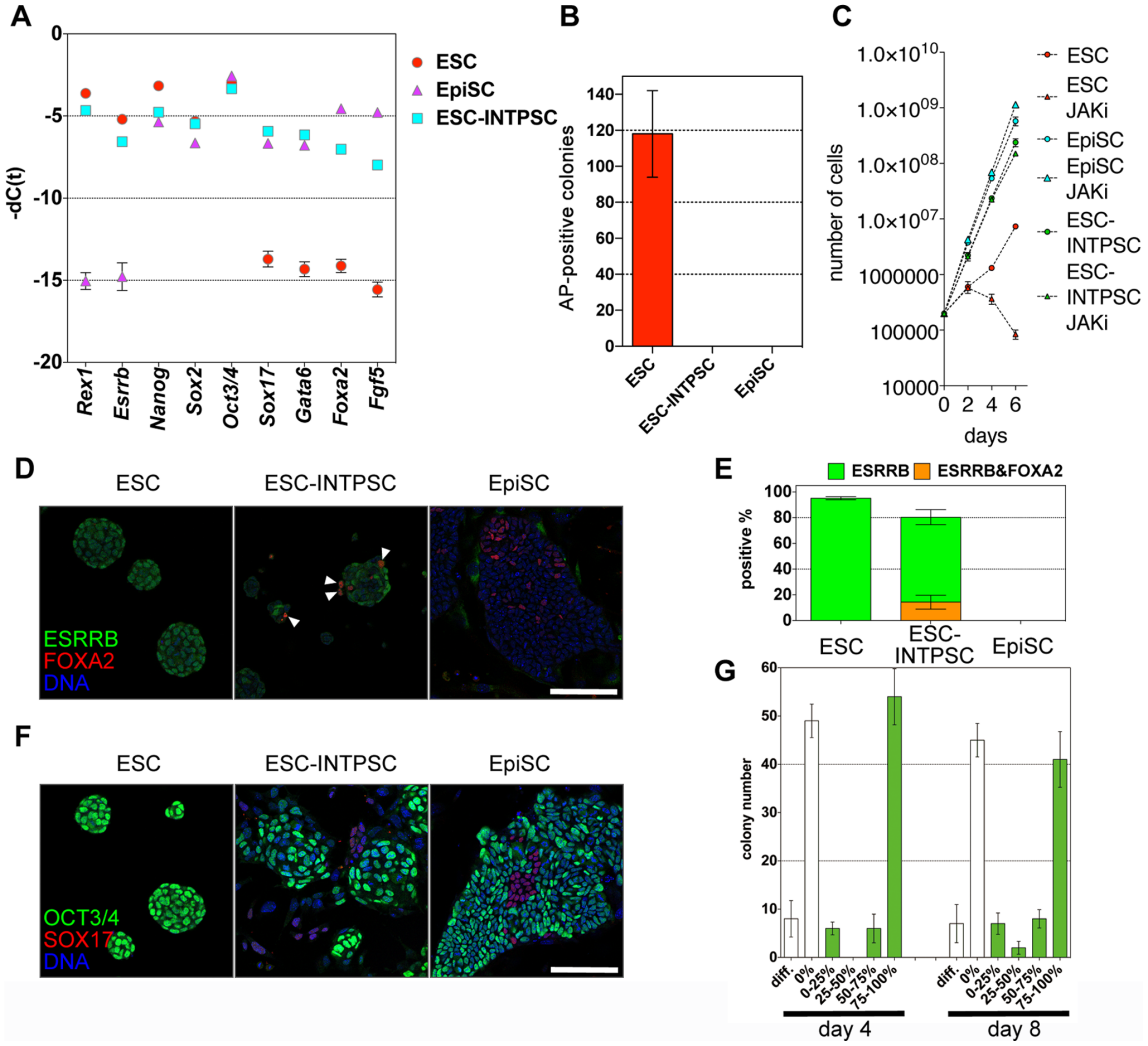
Features of ESC-INTPSCs intermediate between ESCs and EpiSCs. (A) Comparison of gene expression in ESCs, EpiSCs, and ESC-INTPSCs. (B) The number of AP-positive colonies formed in the LIF/2i condition four days after 1000 cells were seeded per dish. (C) Proliferation of ESCs, EpiSCs, and ESC-INTPSCs with or without JAKi. (D) Expression of ESRRB and FOXA2. White arrows indicate ESRRB- and FOXA2-double–positive cells. Scale bar, 100 µm. (E) Percentage of ESRRB-positive or ESRRB- and FOXA2-double–positive cells in ESCs, ESC-INTPSCs, and EpiSCs. (F) Expression of OCT3/4 and SOX17. Scale bar, 100 µm. (G) The number of Oct3/4-GFP–positive or –negative colonies derived from single cells at day 4 and 8.

The morphology of INTPSCs was intermediate between that of ESCs and EpiSCs as shown in Fig. 1C. Therefore, we performed quantitative RT-PCR to analyze the expression of genes that were highly expressed in ESCs and EpiSCs. This analysis revealed that ESC-INTPSCs expressed genes that were also highly expressed in ESCs, including *Rex1* and *Esrrb*; genes highly expressed in EpiSCs, including *Sox17*, *Gata6*, *Foxa2*, and *Fgf5*; and genes highly expressed in both ESCs and EpiSCs, including *Nanog*, *Sox2*, and *Oct3/4* (Fig. 2A). These results indicated that ESC-INTPSCs represent a hybrid state with features of both ESCs and EpiSCs. However, these results were based on analyses of bulk cultures, raising the possibility that the INTPSC cultures were heterogeneous, i.e., characterized by the co-existence of ESC-like and EpiSC-like cells.

To determine whether the co-detection of ESC markers and EpiSC markers in INTPSCs was caused by such heterogeneity, or instead by the presence of bona fide intermediate cells, we performed a series of experiments. First, to determine whether INTPSC cultures contained ESC-like cells, we cultured ESCs, EpiSCs, and ESC-INTPSCs in the LIF/2i condition at clonal density. As expected, ESCs were able to form many alkaline phosphatase–positive colonies. By contrast, neither ESC-INTPSCs nor EpiSCs could form colonies in the LIF/2i condition (Fig. 2B). Thus, INTPSC cultures did not contain ESC-like cells that could grow in the LIF/2i condition. Importantly, as shown in Fig. 1F, most ESC-INTPSCs were OCT3/4-positive regardless of whether they were GOF18 Oct3/4-GFP–positive; however, none of these cells could proliferate in the LIF/2i condition. These results clearly demonstrate that the GOF18 Oct3/4-GFP–positive and –negative states do not correspond to ESC-like and non–ESC-like states, respectively.

To investigate the signaling pathways required for maintenance of self-renewal of INTPSCs, we cultured ESCs, EpiSCs, and ESC-INTPSCs under conditions containing JAK inhibitor (JAKi). ESC-INTPSCs and EpiSCs, but not ESCs, proliferated in this condition, suggesting that INTPSCs do not depend on the LIF–Jak/Stat pathway to proliferate (Fig. 2C). This result also indicates that INTPSCs are distinct from ESCs. Collectively, these results indicate that INTPSC cultures do not contain ESC-like cells.

Next, we performed immunofluorescence staining to examine the expression of ESRRB, a protein that is highly expressed in ESCs, and FOXA2, which is highly expressed in EpiSCs, at the single-cell level. We confirmed that a fraction of OCT3/4-positive EpiSCs express FOXA2, although FOXA2 is known to be an endoderm marker gene (Fig. S1). This experiment revealed that a large proportion of ESCs and ESC-INTPSCs were positive for ESRRB (Fig. 2D and 2E, 95.18±1.20% and 80.40±5.87%, respectively). By contrast, all EpiSCs were negative for ESRRB (Fig. 2D and 2E). These results indicate that most INTPSCs were distinct from EpiSCs. Furthermore, the analysis revealed that a fraction of ESC-INTPSCs, but neither ESCs nor EpiSCs, co-express ESRRB and FOXA2, as determined at the single-cell level (Fig. 2D and 2E, 14.32±3.85% of ESRRB-positive cells). This result clearly demonstrates that INTPSCs are distinct from both ESCs and EpiSCs. Together, these findings show that the features of INTPSCs cannot be explained simply by heterogeneity characterized by the co-existence of ESC-like and EpiSC-like cells.

Additionally, to address whether the detection of endoderm markers in INTPSCs was simply due to the existence of differentiating cells, or was instead due to expression of these markers in undifferentiated INTPSCs, we stained ESCs, EpiSCs, and ESC-INTPSCs with anti-OCT3/4 and anti-SOX17 antibodies. This analysis revealed that a small proportion of OCT3/4-negative INTPSCs expressed SOX17 (Fig. 2F), indicating that the INTPSC cultures contained a small fraction of spontaneously differentiating cells. Taken together, these data show that although the INTPSC culture exhibited some heterogeneity, including cells spontaneously differentiating into endoderm, the hybrid features of INTPSCs were not simply due to heterogeneity characterized by the co-existence of ESC-like and EpiSC-like cells.

Additionally, in order to determine whether INTPSCs fluctuate between Oct3/4-GFP–positive and –negative state, we seeded ESC-INTPSCs in 96-well plates at clonal density. Four and eight days after seeding, we examined colony formation in these cultures. Most of the colonies were either highly GFP-positive or completely GFP-negative (Fig. 2G), indicating that fluctuation of GFP expression did not occur over short timescales.

**Figure 3:**
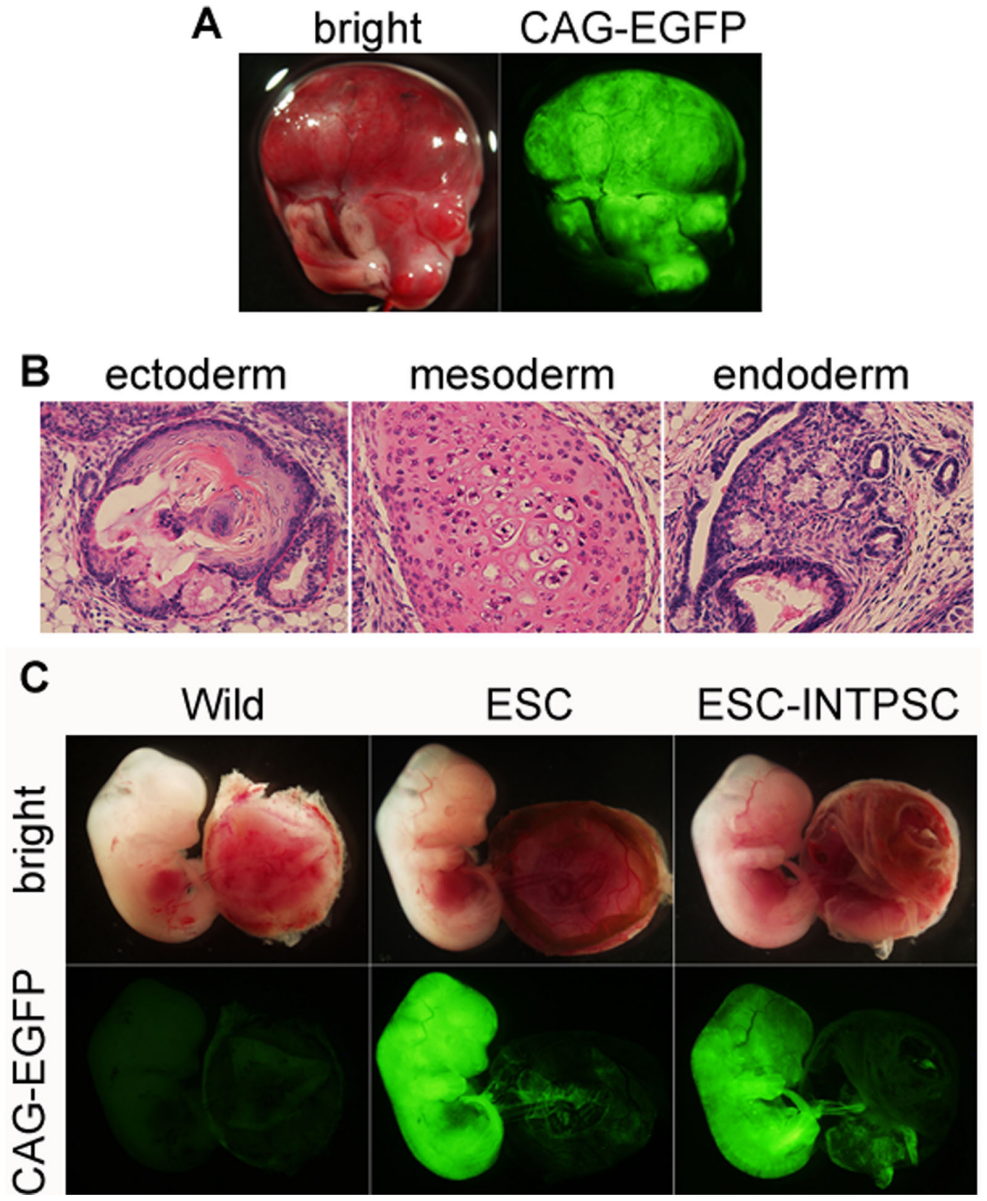
Pluripotency of ESC-INTPSCs. (A) Teratoma formation of ESC-INTPSCs. (B) Differentiation of ESC-INTPSCs into the three germ layers. Left panel shows a keratinocyte-like structure; center panel shows a cartilage-like structure; and right panel shows an intestinal epithelium–like structure. (C) Contributions of ESCs and ESC-INTPSCs to chimeras.

To analyze the pluripotency of these cells, we introduced a constitutively active CAG-EGFP reporter into 129B6F1 ESC-INTPSCs, and then injected the resultant cells into testis capsules of CD-1 immune-deficient mice and CD-1 blastocyst embryos. Six weeks after injection, tumors expressing EGFP developed (Fig. 3A). These tumors contained derivatives from all three germ layers (Fig. 3B), indicating that the ESC-INTPSCs could form teratomas. After injection into blastocysts, chimeras expressing EGFP were detected at E12.5 (Fig. 3C and Table 1), indicating that the ESC-INTPSCs could also contribute to chimeras.

**Table 1:** Chimera production in ESCs and ESC-INTPSCs.

Cell line	Number of chimeric embryos	Number of surviving embryos	Number of transferred embryos
**ESC**	17	59	168
**ESC-INTPSC**	26	48	210

### Generation and Evaluation of iINTPSCs

**Figure 4:**
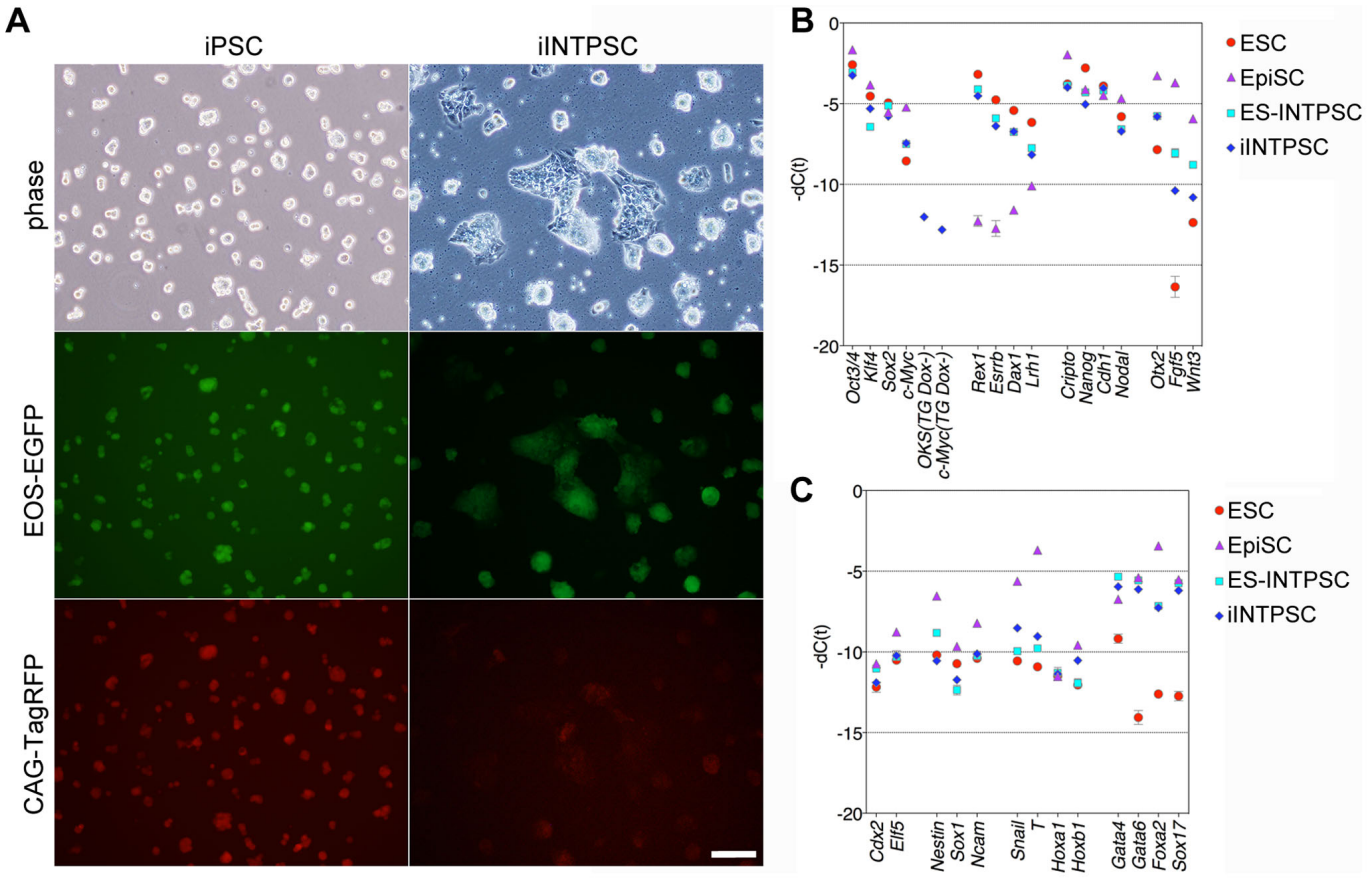
Establishment of iINTPSCs and gene expression in these cells. (A) Morphologies of iPSCs and iINTPSCs. Scale bar, 100 µm. (B) Comparison of endogenous expression levels of the four reprogramming genes, transgenes, ESC-specific genes, and EpiSC-specific genes among ESCs, EpiSCs, ESC-INTPSCs, and iINTPSCs. (C) Comparison of trophectodermal, epidermal, mesodermal, and endodermal marker genes among ESCs, EpiSCs, ESC-INTPSCs, and iINTPSCs.

Next, we tried to generate cell lines from somatic cells under the INTPSC condition using iPSC technology. Recently, we developed a comprehensive system for generation and evaluation of induced pluripotent stem cells using piggyBac transposition and Acr-EGFP [14]. We used this system to efficiently generate and evaluate induced INTPSCs (iINTPSCs). First, we simultaneously introduced five vectors into 129B6F1 mouse embryonic fibroblasts (MEFs). The pCAG-hyPBase vector expresses a hyperactive piggyBac transposase under the control of the CAG promoter [15]. The PB-(CAG-rtTA; EOS-C(3+)-EGFP-IRES-puro) vector expresses reverse trans-activator (rtTA) under the control of the CAG promoter, and contains an EOS-EGFP reporter and a puromycin-resistance gene as markers for pluripotency. The PB-TET-OKS and PB-TET-c-Myc vectors express the reprogramming factors (Oct3/4, Klf4, Sox2, and c-Myc) under the control of a doxycycline (Dox)-inducible promoter. Finally, the PB-(CAG-TagRFP-IRES-hyg; Acr-EGFP) vector expresses TagRFP and a hygromycin-resistance gene under the control of the CAG promoter in most cell types, and also contains a spermatid- and spermatozoa-specific EGFP reporter expressed under the control of the acrosin promoter. After transfection, the cells were cultured in 10% FBS medium containing Dox. Four days after Dox addition, the transfected cells were reseeded on feeder layers. Starting on the next day, the cells were cultured under the INTPSC condition. Ten days after Dox addition, Dox was withdrawn from the culture. After culture for an additional 4 days without Dox, a large number of EOS-GFP–positive colonies appeared. EOS-GFP– and CAG-TagRFP–positive colonies were picked and reseeded on fresh feeder cells. After several passages, the cells were transferred onto feeder-free gelatin-coated dishes. The cells proliferated efficiently under the INTPSC condition and could be propagated by single-cell dissociation, allowing cell lines to be established. These iINTPSC lines expressed TagRFP and expressed EOS-EGFP as a marker of pluripotency (Fig. 4A).

To analyze the molecular state, we analyzed gene expression in ESCs, EpiSCs, ESC-INTPSCs, and iINTPSCs by quantitative RT-PCR. This analysis revealed that ESC-INTPSCs and iINTPSCs expressed genes that are expressed at high levels in ESCs, including *Oct3/4*, *Klf4*, *Sox2*, *Rex1*, *Esrrb*, *Dax1*, and *Lrh1* (Fig. 4B). Genes highly expressed in EpiSCs, including *Otx2*, *Fgf5*, and *Wnt3*, were expressed in ESC-INTPSCs and iINTPSCs at levels intermediate between those in EpiSCs and ESCs (Fig. 4B). Genes highly expressed in both ESCs and EpiSCs, including *Cripto*, *Nanog*, *Cdh1*, and *Nodal*, were also expressed in ESC-INTPSCs and iINTPSCs (Fig. 4B). Trophectodermal, ectodermal, and mesodermal marker genes, including *Cdx2*, *Elf5*, *Nestin*, *Sox1*, *Ncam*, *Snail*, *T*, *Hoxa1*, and *Hoxb1*, were barely expressed in ESC-INTPSCs and iINTPSCs, as in the case of ESCs (Fig. 4C). By contrast, endodermal marker genes, including *Gata4*, *Gata6*, *Foxa2*, and *Sox17*, were highly expressed in ESC-INTPSCs and iINTPSCs, also as in the case of EpiSCs (Fig. 4C). This finding is in agreement with the results of immunostaining assays (Fig. 2D and 2F). Collectively, these results indicate that the gene-expression profiles of ESC-INTPSCs and iINTPSCs are intermediate between those of ESCs and EpiSCs.

**Figure 5:**
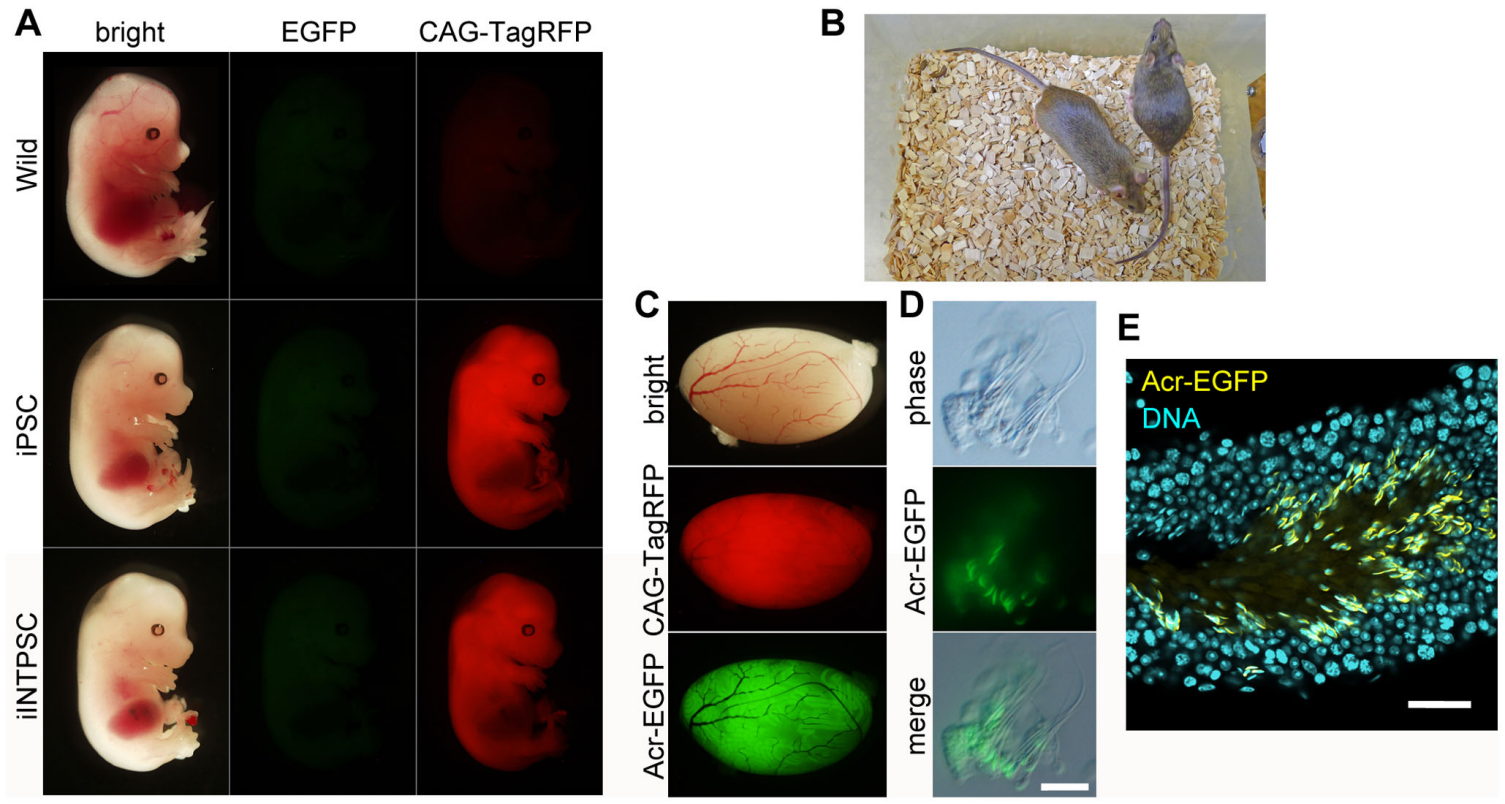
Contribution to chimeras and germ-cell differentiation of iINTPSCs. (A) Chimeric contribution of iPSCs and iINTPSCs. (B) Coat color of adult chimeras of iINTPSCs at 8 weeks of age. (C) Contribution of iINTPSCs to mouse testes. (D) iINTPSC-derived EGFP-positive sperm. Scale bar, 5 µm. (E) EGFP-positive sperm in seminiferous tubules. Scale bar, 50 µm.

To analyze the pluripotency of the iINTPSC lines, we injected these cells into C57BL/6 blastocyst embryos. By visualizing CAG-TagRFP at E14.5, we confirmed that iINTPSCs contributed to chimeric animals, with a contribution ratio comparable to that of iPSCs. No leaky or non-specific expression of EOS-EGFP or Acr-EGFP was observed (Fig. 5A). Coat color of adult chimeras also revealed the ability of the iINTPSCs to contribute to chimeras. Judging from the coats of the chimeric animals, the contribution ratios were high (Fig. 5B and Table 2).

**Table 2:** Chimera production in iPSCs and iINTPSCs.

Cell line	Number of chimeric pups	Number of surviving pups	Number of transferred embryos
**iPSC**	3	12	41
**iINTPSC**	4	14	44

Finally, to evaluate the ability of iINTPSCs to contribute to the germline, we examined the testes of chimeras at the age of 8 weeks. Bright EGFP and TagRFP fluorescent signals were detected in seminiferous tubules of the testes (Fig. 5C). The spermatids and sperm derived from donor iINTPSCs could be distinguished by fluorescence from the Acr-EGFP reporter (Fig. 5D and 5E).

## Discussion

In this study, we established new cell lines that exhibit germline-competent pluripotency using a modified EpiSC culture condition containing the GSK3 inhibitor CHIR99021.

Previous work showed that activation of Wnt signaling can prevent differentiation from ESCs to EpiSCs [10]. However, that study did not examine whether the culture condition used could support establishment of stem cell lines. In this study, we used CHIR99021 as an activator of Wnt signaling; CHIR99021 inhibits GSK3 specifically, resulting in activation of canonical Wnt signaling. Using this small molecule, we were able to establish novel germline-competent pluripotent stem cell lines that differ from both ESCs and EpiSCs.

INTPSC colonies exhibited a dome-shaped morphology, although they were flatter than mouse ESC colonies. In contrast to the situation in EpiSCs, both X chromosomes in INTPSCs were active. The gene-expression profiles of INTPSCs were intermediate between those of ESCs and EpiSCs. For example, *Esrrb* and *Rex1* were expressed in ESCs and INTPSCs but not in EpiSCs, whereas *Fgf5* was expressed in EpiSCs and INTPSCs but not in ESCs. Additionally, endodermal marker genes were expressed in EpiSCs and INTPSCs but not in ESCs. Neither EpiSCs nor INTPSCs could form colonies in the LIF/2i condition unlike ESCs. EpiSCs and INTPSCs, unlike ESCs, were able to proliferate in medium containing JAKi. Furthermore, ESCs and INTPSCs could be propagated by single-cell dissociation, whereas EpiSCs could not. Importantly, ESCs and INTPSCs could generate chimeras efficiently and contribute to the germline, whereas EpiSCs barely contributed to chimeric animals. These results indicate that INTPSCs have properties that are intermediate between those of ESCs and EpiSCs.

Inhibition of GSK3 results in inhibition of Tcf3 and activation of Esrrb in ESCs [16]. Overexpression of Esrrb enables ESCs to maintain self-renewal and pluripotency in the absence of cytokines, and enables reprogramming of EpiSCs into an ESC-like state [17]. Thus, Esrrb is a key factor for maintenance of naïve pluripotency. INTPSCs expressed Esrrb at a level comparable to that in ESCs, and the cells satisfied several criteria for naïve pluripotency. These findings implicate Esrrb in determining the phenotype of INTPSCs.

By contrast, activation of Erk1/2 signaling by FGF stimulation promotes differentiation of ESCs [18]. Stimulation of Activin signaling in ESC cultures induces differentiation into a primitive streak-like state [19], and inhibition of Activin signaling via type I receptors contributes to induction and maintenance of a naïve ESC-like state [20]. Taken together, the opposing effects of CHIR99021 on ESC differentiation may account for the intermediate state of INTPSCs.

In previous work, stem cells were established from mouse blastocysts in the presence of bFGF, Activin A, and BIO (thus, “FAB-SCs”) [21]. BIO is a chemical inhibitor of GSK3, although its specificity is lower than that of CHIR99021; therefore, the culture condition used in that study resembled our INTPSC condition. However, FAB-SCs were not able to contribute to chimeras or form teratomas unless they were stimulated with LIF and BMP4. By contrast, our INTPSCs could efficiently contribute to chimeras and form teratomas even in the absence of such stimulation, suggesting that these cells are distinct from FAB-SCs. Activation of the Wnt pathway by BIO is sufficient to maintain self-renewal of both human and mouse ESCs [22]; however, we could not maintain self-renewal of mouse ESCs with CHIR99021 alone (data not shown), indicating that BIO and CHIR99021 have different effects on pluripotent stem cells.

Similar to INTPSCs, intermediate epiblast stem cells with dual responsiveness to LIF–Stat3 and Activin–Smad2/3 can be established by culturing ESCs in the presence of Activin A [23]. The resultant cells exhibit properties intermediate between those of ESCs and EpiSCs, but cannot contribute to chimeras. Therefore, we propose that there exist several distinct cell states intermediate between ESCs and EpiSCs, and that these states have different capacities for differentiation.

To address the ability of INTPSCs to contribute to the germline, we applied the Acrosin-EGFP system to detect donor-derived sperm, rather than demonstrating germline transmission by mating, for the following reasons: (1) it is not efficient to repeat mating until germline-transmitted pups can be obtained from novel cell lines that are not known to contribute to the germline, because verification of germline transmission by mating is time-consuming: the process takes about 3 months in mouse; (2) when the contribution ratio to the germline is very low, it is difficult to obtain germline-transmitted pups by mating even if the cells actually have germline transmission ability; (3) in chimera formation assays, male donor cells are injected into female host blastocysts at a rate of 50%, and the testes of the resultant bisexual chimeras are often immature, resulting in infertility. Verification of germline transmission by mating is often considered an ideal standard. However, for the reasons described immediately above, use of this method can result in inaccurate assessment of the actual transmission ability. By contrast, the piggyBac system for induction and evaluation of iPSCs that we recently developed permits rapid and efficient investigation (by fluorescence) of the ability of iPSCs to contribute to chimeric animals and the germline [14].

Primed pluripotent stem cells are more difficult to handle than naïve ESCs and iPSCs. Therefore, many researchers have tried to establish naïve stem cell lines in non-rodent species, including human [3], [20], [24], [25]. Our INTPSCs satisfied several criteria for naïve pluripotent stem cells, including activation of X chromosomes and the ability to contribute to chimeras and the germline, but they also met several criteria for primed pluripotent stem cells. Our results suggest that the naïve and primed states are not entirely mutually exclusive, and that the ability of pluripotent stem cells to contribute to chimeras and germline cells by blastocyst injection may depend on the activation state of Wnt signaling.

## Materials and Methods

### Vector Construction

Construction of the PB-TET-OKS and PB-TET-c-Myc vectors was described in previous reports [26]–[28]. To construct PB-(CAG-rtTA_Adv; EOS-C(3+)-EGFP-IRES-puro), an insert from PB-EOS-C(3+)-EiP [26], [29] was introduced into PB-CAG-rtTA_Adv [26], [28]. To construct PB-(CAG-TagRFP-IRES-hyg; Acr-EGFP), an insert from pAcr3-EGFP [30] was introduced into the empty PB vector. The resultant vector (PB-Acr-EGFP) was amplified by PCR, the amplified DNA fragment was cloned into PB-CAG-cHA-IRES-hyg, and an insert from pTagRFP-N1 (Evrogen) was introduced into the resultant vector (PB-[CAG-cHA-IRES-hyg; Acr-EGFP]). To construct pCAG-hyPBase, an insert from pCMV-hyPBase [15] was cloned into pCAGGS [31]. Primer sequences are shown in Table S1.

### Cell Culture

For preparation of ESCs, 129B6F1 blastocyst embryos were collected, seeded on MEF feeder layers, and cultured in ESC medium. ESC medium consisted of 50% Neurobasal medium (Invitrogen) and 50% DMEM/F12 (Invitrogen) containing 0.5×N2 (Invitrogen), 0.5×B27 (Invitrogen), 1% Knockout Serum Replacement (KSR, Invitrogen), 0.05% BSA (Sigma), 0.15 mM 1-thioglycerol (Sigma), 2 mM L-glutamine, 100 U/ml penicillin, and 100 µg/ml streptomycin (Invitrogen) supplemented with 1000 U/ml leukemia inhibitory factor (LIF, Millipore), 1 µM PD0325901, and 3 µM CHIR99021 (2i [2], Stemgent). The cells were cultured on feeder-free gelatin-coated dishes after the first passage.

To adapt ESCs to the INTPSC condition, 5×10^5^ cells were reseeded on feeder-free gelatin-coated dishes, and the medium was replaced with INTPSC medium consisting of 50% Neurobasal medium and 50% DMEM/F12 containing 0.5×N2, 0.5×B27, 1% KSR, 0.05% BSA, 0.15 mM 1-thioglycerol, 2 mM L-glutamine, 100 U/ml penicillin, and 100 µg/ml streptomycin supplemented with 12 ng/ml mouse fibroblast growth factor basic (bFGF, R&D Systems), 10 ng/ml human/mouse/rat Activin A (R&D Systems), and 3 µM CHIR99021. Every 2 days, 5×10^5^ cells were passaged by single-cell dissociation.

EpiSCs were cultured on MEF feeder layers in EpiSC medium consisting of 50% Neurobasal medium and 50% DMEM/F12 containing 0.5×N2, 0.5×B27, 1% KSR, 0.05% BSA, 0.15 mM 1-thioglycerol, 2 mM L-glutamine, 100 U/ml penicillin, and 100 µg/ml streptomycin supplemented with 12 ng/ml mouse bFGF and 10 ng/ml human/mouse/rat Activin A.

For JAKi treatments, ESCs were cultured in LIF ESC medium consisting of Glasgow minimum essential medium (GMEM, Sigma) containing 15% KSR, 0.3% fetal bovine serum (FBS, JRH Biosciences), 1 mM sodium pyruvate (Invitrogen), 1×MEM non-essential amino acids (NEAA, Invitrogen), 0.1 mM 2-mercaptoethanol (2-ME, Millipore), 2 mM L-glutamine, 100 U/ml penicillin, and 100 µg/ml streptomycin supplemented with 1000 U/ml LIF.

For preparation of MEFs, embryos were collected at embryonic day (E) 13.5. After removal of the heads and visceral tissues, the remaining bodies were washed in fresh PBS and trypsinized, and the isolated cells were maintained in Dulbecco’s modified eagle medium (DMEM, Invitrogen) containing 10% FBS, 100 U/ml penicillin, 100 µg/ml streptomycin, and 100 µg/ml primocin (Invivogen).

To establish iINTPSC lines, MEFs were simultaneously transfected with PB-TET-OKS, PB-TET-c-Myc, PB-(CAG-rtTA_Adv; EOS-C(3+)-EGFP-IRES-puro), PB-(CAG-TagRFP-IRES-hyg; Acr-EGFP), and pCAG-hyPBase [23], [26], [27]. The transfected cells were cultured in 10% FBS–DMEM. One day after transfection, the medium was replaced with GMEM containing 10% FBS, 1 mM sodium pyruvate, 1×NEAA, 0.1 mM 2-ME, 2 mM L-glutamine, 100 U/ml penicillin, and 100 µg/ml streptomycin (10% FBS medium) supplemented with 1.5 µg/ml doxycycline (Dox, Sigma). Four days after Dox addition, 1×10^5^ cells were reseeded on an SNL feeder layer, and the medium was replaced with INTPSC medium the next day. Ten days after Dox addition, Dox was withdrawn. After an additional 4 days of culture without Dox, CAG-TagRFP– and EOS-EGFP–double-positive primary colonies were picked, enzymatically dissociated, and transferred onto a fresh SNL feeder layer in 48-well plates. After several passages, the cells were transferred onto feeder-free gelatin-coated dishes and propagated by single-cell dissociation.

### Immunofluorescence Staining and AP Staining

For immunofluorescence analysis, cells were fixed with 4% paraformaldehyde. After washing with PBS, cells were blocked with 0.5% normal goat or donkey serum containing 0.1% Triton X-100, and then treated with primary antibodies. Primary antibodies included anti-Oct3/4 rabbit polyclonal antibody (1∶500; PM048, MBL), anti-Nanog rabbit polyclonal antibody (1∶500; a gift from S. Yamanaka), anti-H3K27me3 rabbit polyclonal antibody (1∶250; 07–449, Millipore), anti-Esrrb mouse monoclonal antibody (1∶500; PP-H6705-00, Perseus), anti-Foxa2 goat polyclonal antibody (1∶1000; sc-9187, Santa Cruz Biotechnology), and anti-Sox17 goat polyclonal antibody (1∶1000, AF1924, R&D Systems). Alexa Fluor 594–conjugated goat anti-mouse IgG (1∶500, Invitrogen), Alexa Fluor 568–conjugated goat anti-rabbit IgG (1∶500, Invitrogen), Alexa Fluor 568–conjugated donkey anti-goat IgG (1∶500, Invitrogen), Alexa Fluor 488–conjugated goat anti-rat IgG (1∶500, Invitrogen), Alexa Fluor 405–conjugated goat anti-mouse IgG (1∶500, Invitrogen), and Alexa Fluor 405–conjugated goat anti-rabbit IgG (1∶500, Invitrogen) were used as secondary antibodies. Nuclei were stained with 1 µg/ml DAPI (Sigma) or 1 µM TO-PRO-3 iodide (Invitrogen). Alkaline phosphatase staining was performed as described previously [32].

### Quantitative RT-PCR

Total RNA was extracted from cells using RNeasy Mini kits (Qiagen). For reverse transcription, ReverTra Ace (Toyobo) and oligo (dT)_20_ primer were used. For real-time PCR, Power SYBR Green PCR Master Mix (Applied Biosystems) was used. Transcript levels were determined in triplicate reactions and normalized against the corresponding levels of *Gapdh*. Primer sequences are shown in Table S1.

### Chimera Production

To generate chimeric animals, 129B6F1 ESCs and ESC-INTPSCs were injected into CD1 embryos at the blastocyst stage. The blastocysts were transferred into the uterine horns of pseudopregnant mice. Chimerism of live embryos was judged by GFP fluorescence and eye color at E12.5.

iPSCs and iINTPSCs were injected into C57BL/6 embryos at the blastocyst stage. The blastocysts were transferred into the uterine horns of pseudopregnant mice. Chimerism of live embryos and pups were judged by RFP fluorescence, eye color, and coat color. The contribution of Acr-EGFP–positive cells into the lumina of seminiferous tubules was analyzed by fluorescence microscopy at the age of 8 weeks.

### Animal Ethics Statement

All animal experiments conformed to our Guidelines for the Care and Use of Laboratory Animals and were approved by the Institutional Committee for Laboratory Animal Experimentation (RIKEN Kobe Institute).

## Supporting Information

Figure S1 **Expression of OCT3/4 and FOXA2.** White arrows indicate OCT3/4- and FOXA2-double–positive cells. Scale bar, 100 µm.
(PDF)

Table S1 **Primer sequences.**
(XLSX)
