# Supplementary material for: A Modified EpiSC Culture Condition Containing a GSK3 Inhibitor Can Support Germline-Competent Pluripotency in Mice
Source: PLoS One. 2014 Apr 15;9(4):e95329. doi: 10.1371/journal.pone.0095329 (PMC3988182; doi:10.1371/journal.pone.0095329)
Supplement: Figure S1 — Expression of OCT3/4 and FOXA2. White arrows indicate OCT3/4- and FOXA2-double–positive cells. Scale bar, 100 µm. (PDF) [file pone.0095329.s001.pdf]

Figure S1 Expression of OCT3/4 and FOXA2

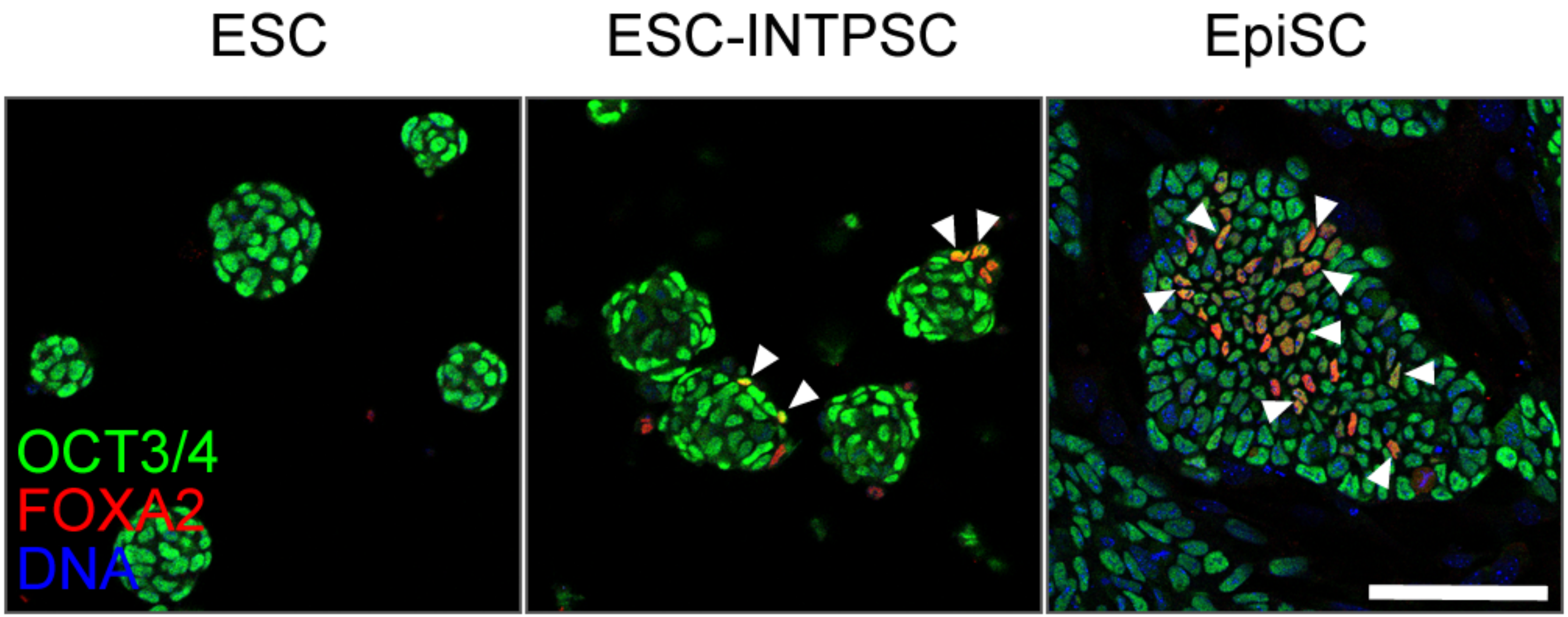

White arrows indicate OCT3/4- and FOXA2-double–positive cells.

Scale bar, 100 μm.
